# Novel Coronavirus (COVID-19) and Dentistry–A Comprehensive Review of Literature

**DOI:** 10.3390/dj8020053

**Published:** 2020-05-21

**Authors:** Poyan Barabari, Keyvan Moharamzadeh

**Affiliations:** 1 Eastman Dental Institute, University College London, London WC1X 8WD, UK; p.barabari@ucl.ac.uk; 2 School of Clinical Dentistry, University of Sheffield, Sheffield S10 2TA, UK

**Keywords:** coronavirus, COVID-19, SARS CoV-2, dentistry, dental practice

## Abstract

The novel coronavirus (COVID-19) pandemic has become a real challenge for healthcare providers around the world and has significantly affected the dental professionals in practices, universities and research institutions. The aim of this article was to review the available literature on the relevant aspects of dentistry in relation to COVID-19 and to discuss potential impacts of COVID-19 outbreak on clinical dentistry, dental education and research. Although the coronavirus pandemic has caused many difficulties for provision of clinical dentistry, there would be an opportunity for the dental educators to modernize their teaching approaches using novel digital concepts in teaching of clinical skills and by enhancement of online communication and learning platforms. This pandemic has also highlighted some of the major gaps in dental research and the need for new relevant knowledge to manage the current crisis and minimize the impact of such outbreaks on dentistry in the future. In conclusion, COVID-19 has had many immediate complications for dentistry of which some may have further long-term impacts on clinical practice, dental education and dental research.

## 1. Introduction

A highly infectious pneumonia started to spread in Wuhan, China, from 12 December 2019 [1]. In early January 2020, the officials announced the novel coronavirus (COVID-19) as the causative pathogen of the disease [2]. This novel viral pneumonia was named “Corona Virus Disease (COVID-19)” by the World Health Organization (WHO). “SARS CoV-2” was also the given name for this novel coronavirus by the International Committee on Taxonomy of Viruses (ICTV) [3].

Soon, it turned into one of the toughest public health challenges in the modern world having spread in over 200 countries across the globe. On 30 January 2020, the WHO declared the COVID-19 outbreak as a public health emergency of an international scale [4,5].

COVID-19-related clinical symptoms can vary from case to case but the most common symptoms are fever, continuous dry cough and myalgia or fatigue and in more severe cases, abnormal chest computed tomography (CT) scan findings such as bilateral and peripheral ground-glass and consolidative pulmonary opacities have been reported [6]. Some evidence highlighted the production of sputum, headache, diarrhea and hemoptysis amongst the less common clinical symptoms [7,8,9]. These symptoms are slightly different from those of severe acute respiratory syndrome (SARS) caused by SARS coronavirus which was widely spread in early 2000s. The differences between SARS and COVID-19 are hidden in their transmissibility and severity pyramids. The transmissibility rate of COVID-19 is reported to be higher than that of SARS [10]. Additionally, in comparison with SARS, a larger population of COVID-19 positive patients demonstrate mild or no symptoms which makes it challenging to diagnose the patients clinically during the incubation period, and therefore, spread of infection can occur at an accelerated rate [11]. It has been indicated that infectious pathogens of COVID-19 had been transmitted via human contact and led to the widespread viral outbreak [2,12]. Although loss of sense of smell and taste were not initially evidenced as symptoms of COVID-19, recent reports suggest that olfactory and gustatory disorders were prevalent symptoms in COVID-19 patients in Europe [13]. Additionally, some new evidence on the impact of COVID-19 on the central nervous system suggests that SARS-CoV-2, like other coronaviruses such as SARS-CoV and MERS-CoV, could target the central nervous system, possibly infecting neurons in the nasal passage and disrupting the senses of smell and taste [14]. Oral manifestations [15] and cutaneous lesions [16] associated with COVID-19 have been reported in adults and more recently children [17]. There is some clinical evidence that COVID-19-positive patients with a history of cardiac risk factors [18] and established cardiovascular disease (CVD) showed more vulnerability to developing severe symptoms and poorer clinical outcomes [9,19]. The significance of CVD in prognosis of such patients was established in a cohort of 191 patients, where 30% had hypertension and constituted 48% of non-survivors, whereas CVD was present in 8% who constituted 13% of non-survivors [20]. A meta-analysis of 1527 patients with COVID-19 reported an overall case fatality rate of 2.3% in the entire cohort. However, this rate was significantly higher in patients with hypertension, diabetes, and CVD with 6, 7.3, and 10.5 percent, respectively [21,22].

In 1968, a Nature publication by Almeida et al. first described a newly discovered single-stranded RNA virus with a diameter of 120 nanometers [23]. They decided to name this new group of viruses “corona virus” due to their appearance under the electron microscope. The distinguishing fringe of projections on the outer surface of the virus reminded the scientist of solar corona. Coronaviruses are prone to mutation and recombination, and therefore, around 40 different variations of coronaviruses have been recognized mostly infecting human and non-human mammals and birds [24].

Some evidence suggests that the pathogen of COVID-19 originated in some species of bats first, and it was then spread to intermediate hosts such as wild dogs, snakes and pangolins. The spread to human is thought to have happened via contaminated meat products from traditional wildlife market in Wuhan [25,26]. 

The aim of this article was to review the available literature on the relevant aspects of dentistry in relation to COVID-19 and to discuss potential impacts of the novel coronavirus pandemic on clinical dentistry, dental education and research.

## 2. Potential Routes of Transmission 

Novel coronavirus can be transmitted directly through cough, sneeze and inhalation of virus-containing droplets and microdroplets from infected individuals. It is also transmittable via contact with oral, nasal and eye mucous membranes [27]. Clinical manifestations of the COVID-19 have not been associated with any visual impairment; however, some evidence suggests that eye exposure can provide a viable transmission route to the host’s body, and therefore, its spread is not limited to the respiratory tract [7,28]. A case report from Germany has provided further evidence regarding possible transmission of COVID-19 through contact with asymptomatic patients [29].

There are also several studies indicating that transmission of COVID-19 may become airborne during aerosol generating procedures (AGP) [30]. In a case study in the United States, real-time polymerase chain reaction (RT-PCR) tests of nasopharyngeal and oropharyngeal swabs and stool samples collected one week after patient’s illness detected RNA of the virus and tested positive for COVID-19 [31]. However, more conclusive evidence is needed to confirm the fecal-oral route for transmission of the novel coronavirus. 

## 3. Spread of COVID-19 in Dental Setting 

As evidence suggests, direct passage and spread of novel coronavirus through respiratory droplets and fomite are very common [28,32]. Additionally, incubation period can vary between 7 to 24 days, which in some cases demonstrates no clinical symptoms [8,33]. Therefore, both patients and dental professionals are at a bilateral risk of being exposed to viral pathogens that can be transmitted through the oral cavity and respiratory tract during dental visits. Dental procedures by their very nature have a high risk of COVID-19 infection due to face-to-face communication with patients and the dental team. In addition, frequent contamination with saliva, blood and other body fluids as well as the use of sharp and high-speed rotary instruments magnifies the risk of infection in dental practices. A published report suggests that transmission of COVID-19 pathogen can also happen via inhalation of airborne viruses suspended in the dental surgeries for long hours [34]. Clinical studies indicate that most of the dental procedures involving use of rotary handpieces generate considerable amount of contaminated and potentially infectious aerosol and droplets [35].

Currently, there is not a practical solution to avoid generation of aerosols mixed with patient’s blood and saliva, and this creates great concerns regarding transmission of COVID-19 pathogenic agents during AGPs to the dental team and patients. Moreover, aerosol can stay airborne for an extended length of time entering the patients’ and dental professionals’ body through respiratory tract. The aerosol can also settle on the surfaces of the dental surgery and instruments making cross-contamination between the attendees to the dental surgery highly possible in the absence of effective and rigorous cross-infection control protocols [36].

## 4. Immediate Impact of COVID-19 on Dental Practice 

After announcing COVID-19 as a pandemic by the WHO, The New York Times magazine published an article ranking the health professions at the highest risk of COVID-19 infection, amongst which dental professionals occupied the top of the ranking [37]. The American Dental Association (ADA) announced that COVID-19 transmission was increasing in all states across the US and that all dentists should limit their dental care to only emergency cases. It was imperative that appropriate Personal Protective Equipment (PPE) is utilized to minimize the risk of transmission during emergency dental treatments [38]. On 18 March 2020, in the light of the UK moving into the “delay” phase of its COVID-19 response, the Chief Dental Officer (CDO) suggested a number of preventative measures including displaying communication posters on COVID-19, adopting mechanisms to establish potential patients with symptoms prior to dental visits, ceasing dental treatment for individuals with COVID-19, reducing the numbers of routine check-ups and avoiding AGPs, bearing in mind the potential risk of seeing asymptomatic patients in the delay phase [39]. On 25 March 2020, the CDO of England suggested to put a pause on all non-urgent dental treatments. All dental practices were advised to run a remote service, carry out patient triage for those in urgent need of emergency dental care and provide appropriate advice, analgesia and antimicrobial treatment. Arrangements were also made for creating Local Urgent Dental Care hubs across the UK where the patients were referred if their problem was not manageable remotely and via advice [40]. 

These dental emergency categories included [41]:

- Severe and uncontrolled pain;
- Spreading, recurrent or continuing infection;
- Avulsed permanent tooth;
- Severe trauma.

It was also advised that all community outreach activities such as oral health improvement programs should be stopped until advised otherwise.

UK General Dental Council (GDC) released some guidelines for remote consultation and prescription. It was suggested that patient safety must be the priority, and the identity of each patient must be checked and verified. Dentists should be able to collect sufficient information regarding patient’s health and conditions in order to be able to prescribe the medication safely. It is also important to identify vulnerable patients and take appropriate steps to protect them including obtaining valid and informed consent and following relevant mental capacity law and codes of practice by all prescribing professionals. This guideline also provided further details regarding the information that is required to be provided to the patients for each e-Consultation and prescription, including note keeping that must be clear and should justify the clinician’s decisions [42]. One of the limitations of the remote service was that the General Pharmaceutical Council produced a list of medicines that could not be prescribed remotely unless some safeguarding measures were implemented. This document explained that pharmacies based in England, Scotland and Wales may not supply these categories of medicines without having an assurance that these safeguards are in place [42].

### 4.1. PPE for Treating COVID-19 Patients 

In the UK, patients who had been tested positive for COVID-19 were not expected to be treated in general dental practices. Instead they were referred to their Local Urgent Dental Care centers. Guidelines regarding PPEs for health and social care workers were being regularly updated by the Public Health England (PHE) as new evidence emerged about COVID-19 [43,44]. The guidance released on 7 April 2020 by PHE included the following statement:

“*A long-sleeved disposable fluid repellent gown (covering the arms and body), a filtering face piece class 3 (FFP3) respirator, a full-face shield or visor and gloves are recommended during AGPs on possible and confirmed cases, regardless of the clinical setting. Subject to local risk assessment, the same precautions apply for all patients regardless of case status in contexts of sustained COVID-19 transmission. Where an AGP is a single procedure, PPE is subject to single use with disposal after each patient contact or procedure as appropriate*”.

England NHS advised that all COVID-19 related waste must be regarded as infectious clinical waste (EWC code 18-01-03*). Therefore, it must be stored in a UN-approved orange waste bags in line with the safe management of healthcare waste (HTM07-01).

### 4.2. Potential Role of Dentists and Patients in Reducing the Spread of COVID-19 

#### 4.2.1. Strict Protocol for Patient Screening 

Dental professionals must be able to screen and identify potential high-risk COVID-19 patients to prevent the spread of the infectious disease. No routine dental treatment should be carried out on patients at the early stages of infection, and these patients should be encouraged to quarantine and self-isolate themselves [27]. The first screening measure would be taking the body temperature of each patient using a contact-free forehead thermometer. Patients should fill in a questionnaire answering questions to determine if they have had COVID-19 symptoms such as fever, persistent cough and difficulty breathing within the past two weeks. Any contacts with individuals who had tested positive for COVID-19 should be recorded. Patients should also report if they have had contact with at least two people who demonstrated fever or respiratory symptoms within the last two weeks. The social history and any participation in gatherings and meetings need to be noted as well. 

If the results of screening indicate potential for COVID-19 infection, the patient should be advised to self-isolate at home and contact their COVID-19 care center if the symptoms get worse [27].

#### 4.2.2. Hand Hygiene 

Hand washing is one of the most frequently emphasized measures by WHO and healthcare authorities to limit the spread of coronavirus. Reinforcement of good hand hygiene for both patients and dental professionals is vital as appropriate hand washing protocol may not be followed on some occasions which can create unnecessary challenges for infection control during a pandemic. It has been suggested that dental professionals must wash their hands before examining a patient, prior to any dental procedure, after contacting the patient, and after touching any equipment and surrounding surfaces without disinfection. Hands must be washed also after any direct contact with oral mucosa, wounds or damaged skin, blood, body fluid, saliva and excreta. Dental professionals must avoid touching their own eyes, nose and mouth until it safe to do so [27].

Use of alcohol-based hand rubs with at least 60% ethanol or isopropanol has also been documented as a simple and effective cross-infection control technique which can inactivate enveloped viruses, including coronaviruses [45].

#### 4.2.3. Personal Protective Measures for Dental Professionals 

Previously, SARS coronavirus infection affected a large number of medical and dental professionals in the hospital setting [46], and therefore, it is extremely important to implement effective PPE measures in dental care centers during the COVID-19 outbreak to ensure safety of both patients and the dental healthcare professionals [27]. As mentioned in the previous section, the use of barrier protection, such as full-length fluid repellent gowns, FFP3 respirators, full-face shield or visor and gloves, is highly recommended especially during AGPs on high risk patients.

#### 4.2.4. Mouth Rinse before Dental Procedures 

Generation of aerosol during most dental treatments is almost unavoidable. Therefore, it is important to reduce the viral load in the droplets and aerosols by preventative measures such as pre-operative use of antiseptic mouth rinses. The most commonly used mouth rinses in dental practices are chlorhexidine and essential oil-based products which may not be as effective as 1% hydrogen peroxide or 0.2% povidone iodine as COVID-19 pathogen is more vulnerable to oxidation [27]. Povidone iodine solution has demonstrated 99.99% activity when used against enveloped and non-enveloped viruses such as influenza, Ebola, MERS and SARS coronavirus [47], and it has strong bactericidal and viricidal properties against pathogens, causing oral and respiratory tract infections. The available evidence provides a strong rationale for the safe use of povidone iodine oral solution as a protective oropharyngeal hygiene measure for individuals at high risk of exposure to oral and respiratory pathogens [48].

#### 4.2.5. Rubber Dam Isolation 

One of the simplest and practical ways in reducing contamination from the oral cavity and achieving effective moisture control is application of rubber dam. Rubber dam isolation is proved to be effective in minimizing production of saliva and blood-contaminated aerosols, especially during the procedures carried out with the use of high-speed handpieces and ultrasonic instruments. Evidence suggests that the use of rubber dam resulted in a 70% reduction of airborne particles within approximately 3-foot diameter of the operational field [49]. It would be also advantageous to disinfect the operative area with povidone iodine or peroxide solution after rubber dam placement. A high-volume suction can also be useful along with the rubber dam to further minimize the risk of contamination. When application of rubber dam is not feasible, manual instruments such as excavators and hand scalers should be utilized to keep aerosol generation at a minimal level [50].

#### 4.2.6. Anti-Retraction Handpiece 

The use of an anti-retraction handpiece results in a significant reduction in the backflow of bacteria and Hepatitis B virus (HBV) from oral cavity into the tubes of the handpiece and the dental unit in comparison with a handpiece without anti-retraction function. The ordinary handpieces can aspirate and eject the contaminated fluids during dental procedures and as a result, oral flora including bacteria and viruses may contaminate the air and water tubes within the dental unit and cause cross-infection. Thus, utilization of anti-retraction handpieces is advised and encouraged especially during the pandemic of COVID-19 [51]. 

#### 4.2.7. Strict Disinfecting Protocol for Clinical Environment 

It is crucial that medical and dental teams follow an effective and strict disinfection protocol for both clinical and communal areas. All surfaces in the clinical areas must be cleaned and disinfected to the highest standard according to the local guidelines and requirements. The communal areas and public facilities must be cleaned and disinfected on a regular basis, which will include total disinfection of all door handles, chairs, desks, touch screens and monitors. If there is a lift in the building, it should be disinfected frequently, and all lift users should be encouraged to wear masks and avoiding direct contacts with any buttons in the lift [27]. 

Installation of enhanced air ventilation systems in healthcare premises can also help to facilitate removal of airborne pathogens from clinical environments and reduce the risk of infection [52].

#### 4.2.8. Clinical Waste Management 

Clinical waste should be stored in a safe temporary storage area, and all reusable instruments and items should be pre-treated, cleaned, sterilized and properly stored in accordance with the local protocols. The clinical waste generated after treatment of COVID-19 positive patients must be regarded as infectious clinical waste and stored in clinical waste bags within a designated area. The surface of the package bags should be marked and disposed according to the local regulations and requirement for the management of medical waste [27]. 

## 5. COVID-19 Testing 

There are various methods available for COVID-19 testing, and the decision to carry out a test on suspected individuals should be made based on clinical symptoms and epidemiological factors. It would be beneficial if dental practices were provided with fast COVID19 detection kits in order to test the high-risk and suspicious patients. This way, they can take necessary precautions in reducing the spread of the virus and also help the national healthcare system to track the possible infected cases and gather data regarding the number of infected cases in the country.

### 5.1. RT-PCR

As per WHO guideline, the RT-PCR test should be done in asymptomatic or mildly symptomatic patients and those who have had contact with COVID-19 positive cases. Screening protocols and local guidelines for the patient evaluation should be followed and complied with. A crucial step in management of outbreak is fast collection of samples and testing of the suspicious cases utilizing nucleic acid amplification test (NAAT) like RT-PCR [53]. The RNA test for detection of SARD-CoV-2 genetic signature is used in this method. A swab is normally utilized to gather specimen from inside the nose or posterior part of throat. The result of nucleic acid testing via PCR, which is capable of detecting even a small amount of RNA, can be communicated within hours. The test is highly sensitive and specific, but its accuracy depends on the quality of the samples provided. The limitation of PCR testing is that it can only identify patients with ongoing infection, and those in the recovery phase may not have detectable virus, hence, the test could be negative [54].

For the purpose of PCR testing, specimens should be collected from upper respiratory tract using nasopharyngeal and oropharyngeal swabs. In lower respiratory tract, specimen should be collected from the sputum (if present) or endotracheal aspirate or bronchoalveolar lavage in severely symptomatic patients. Having detected COVID-19 virus in the blood and stools of infected patients, blood and stool collected from suspicious cases can also be used for testing. The duration and frequency of shedding of COVID-19 virus in stool and potentially in urine is unknown. In deceased patients, autopsy material from lung tissue would be a useful specimen, and in recovering patients, paired serum can be used to define cases as serological assays appear [55].

Collection, handling and packaging of specimens for virus detection is vital, and specimens must be delivered to the laboratory as soon as possible. If the specimen can be delivered quickly to the nominated laboratory, they can be stored and shipped at 2–8 °C. In the case of delay, the use of viral transport medium is necessary, and specimen should be frozen at −20 °C or ideally −70 °C. It should be noted that repeated freezing must be avoided [53].

### 5.2. Serological or Antibody Testing 

Another method of investigation in an ongoing pandemic is serological survey of cases with negative NAAT assays but strong epidemiological link to COVID-19. In these cases, paired serum samples could support the diagnosis once validated serology tests are available. The challenge of this test could be the cross reactivity with other coronaviruses [56]. This method tests whether a suspected individual has been infected by COVID-19 and has produced antibodies. The immunological reactions to the SARS-CoV-2 can take several weeks to happen, and some studies show that antibodies to COVID-19 may take 14 days to appear. Therefore, a serology test before this period may result in an unhelpful negative [54].

Antibody test requires a blood sample from the patient, and it will look for the evidence of the virus in the body by detecting antibodies produced by the immune system in reaction to the infection. Body’s first reaction to an infection is in the form of IgM as it appears in the blood approximately within 5 to 10 days post-infection and reaches its peak at day 21 of the infection. The time-frame is vital, because if a suspicious patient develops COVID-19 symptoms, it takes about a week for their body to produce anti-COVID-19 IgM. Although for the novel coronavirus there is evidence of IgM’s presence in the blood within a day of developing symptoms, it will not be completely reliable as large quantities of IgM are not available for detection. The PCR test is reported to have a 66.7% detection rate within the first week of infection whereas antibody test has 38.3% detection rate. Therefore, the most reliable test for an early infection would be combination of antibody and PCR swab from suspicious patients. The combining of the tests will result in 98.6% detection rate within the first 5.5 days post-infection [57]. Another type of antibody is IgG, which proves that a person has had the viral infection, and the body is now immune to the pathogen. This test also needs blood sample from patients, and IgG can be detected in the blood approximately 14 days after infection. If the patient becomes re-exposed to the virus, the IgG level is rapidly raised within 48 hours to fight the virus and prevent a new infection. Therefore, if the serology test of a patient only detects IgM, it indicates that they are within the first week of infection. The presence of both IgM and IgG suggests that patient should be in their first month of infection and theoretically immune to the virus and re-infection [58].

IgA is another parameter that can be measured to aid the detection of infected COVID-19 cases. It is present in serum, nasal mucus, saliva, breast milk and intestinal fluid, and it consists of around 10 to 15 percent of human immunoglobins. Gou et al. examined the humoral response of the host immune system including IgA, IgM and IgG responses by testing 208 plasma samples from 82 confirmed and 58 probable cases (qPCR negative but with clinical symptoms) utilizing an ELISA-based assay. The median duration of IgM and IgA detection were 5 days while IgG was detected on 14 days of clinical manifestation of symptoms with a positive rate of 85.4%, 92.7% and 77.9%, respectively. The findings suggested that the positive rates for IgM were 75.6% and 93.1% in confirmed and probable cases, respectively. It concluded that the detection efficiency of IgM ELISA method was higher than that of qPCR after 5.5 days of the symptoms’ onset. The combination of IgM ELISA assay and PCR test significantly increased the positive detection rate to 98.6% for each patient. This number is 51.9% with a single PCR test. Thus, it can be concluded that the immune response of the host body can be utilized to increase the detection rate and diagnosis of COVID-19 cases [59].

### 5.3. Medical Imaging 

The review of the relevant literature in medical imaging suggests that chest radiographs have no diagnostic value in early stages of the infection. However, CT scan may present some findings even before the onset of symptoms [60]. Bilateral multilobar ground-glass opacificities associated with a peripheral asymmetric and posterior distribution is the typical feature of the CT in COVID-19 positive cases. As the infection develops subpleural dominance, crazy paving and consolidation can be observed in most cases. A comparative study conducted in Wuhan, the origin of COVID-19, suggests that CT is significantly more sensitive than PCR test; however, it is less specific as many of its imaging characteristics overlap with other types of pneumonia [61]. American College of Radiology also recommends that CT imaging should not be used as a first-line test to screen potential COVID-19 cases [62]. The Centre for Disease Control in the U.S. recommendation is to utilize PCR testing for initial screening of suspected cases [63]. In the UK, the Royal College of Radiologists announced that the use of additional chest CT to assess patients for the presence of likely COVID-19 infection may have a role in stratifying risk in patients presenting with acute symptoms and requiring a CT of abdomen, particularly those needing emergency surgery. In the absence of rapid access to other forms of COVID-19 testing, this is appropriate if it will change the management of the patient. However, a negative scan would not exclude COVID-19 infection, and as with all other current advice, this may change in the future [64]. 

## 6. Treatment of COVID-19 

Potential interventions including pharmacologic treatments for novel coronavirus (COVID-19) disease have been comprehensively and systematically reviewed in two recently published articles [65,66]. 

Below is the summary of these potential therapeutic interventions:

- General supportive treatments such as vitamins (A, B, C, D and E), omega-3 polyunsaturated fatty acids (PUFA), selenium, zinc and iron.
- Coronavirus protease inhibitors including chymotrypsin-like (3C-like) inhibitors (e.g., *Cinanserin* and *Flavonoids)* and papain-like protease (PLP) inhibitors3 such as *diarylheptanoids.*
- Monoclonal antibody (mAb). There is some evidence showing that mAbs can target weak sites on the S protein of virus and neutralize SARS-CoV-2 in cell culture [67].
- Chloroquine and Hydroxy-Chloroquine. They act by changing the pH of endosomes and believed to prevent viral entry, transport and post-entry events; however, their mechanism of action has not been fully discovered [68].
- Emodin is an anthraquinone compound widely used in Chinese herbal medicine. In vitro evidence shows that it blocks the S protein and blocks angiotensin-converting enzyme-2 (ACE2) interaction in a dose-dependent manner. It has inhibited the infectivity of S protein-pseudotyped retrovirus to Vero E6 cells.
- Promazine is an antipsychotic drug and demonstrates a similar structure to emodin on its spike protein binding site structure that contributes to the replication suppression of SARS-CoV. However, promazine has displayed more potent spike-protein-mediated ACE2 binding inhibition than emodin [69].
- Nicotianamine can be found in some plants and in soybean in high concentration. It has been shown as a potent inhibitor of human ACE2 with an IC50 of 84 nM [70]. Since dietary phytochemicals as naturally occurring compounds display a wide safety profile and less pharmacological side effects, nicotianamine constitutes a potential candidate drug for ACE2 inhibition and thus blockade of SARS-CoV-2 cell entry [71].
- Soluble ACE2 receptor. There is some initial evidence to propose that the soluble form of ACE2 which lacks the membrane anchor may prevent binding of the viral particle to the surface-bound receptors by acting like competitive interceptor. Some in vitro evidence suggests that fusion of ACE2 to Fc protein of immunoglobulin can neutralize SARS-CoV-2 [72,73].
- Immuno-enhancers including interferons, intravenous gammaglobulin, thymosin α-1, thymopentin, levamisole and cyclosporine A.
- Antiviral medications such as ribavirin, lopinavir (LPV)/ritonavir (RTV) (Kaletra), Remdesivir, nelfinavir, arbidol and nitric oxide
- Adjunctive therapies including α-lipoic acid, mucroporin-M1, estradiol and phytoestrogen

A large number of clinical trials are currently underway in many countries globally to investigate the suitability of some of these interventions individually or as combination therapies against COVID-19 disease. Multiple vaccine development strategies such as virus vaccines, recombinant protein subunit vaccines and nucleic acid vaccines are also being evaluated for their safety and efficacy [74].

## 7. Impact of COVID-19 on Dental Education 

Immediate effect of COVID-19 on the education sector was noticed very soon after the announcement of the need for “social distancing” and minimizing all face-to-face communication including teaching and educational activities. Although direct and open communication with peers, tutors and the educational team is proved to increase the level of trust and cooperation [75], most of the regulatory bodies across the world advised the higher education institutions to prioritize safety and wellbeing of their students and staff by ceasing all on campus teaching sessions. Subsequently, all dental schools and post-graduate teaching providers stopped their routine face-to-face educational sessions and hands-on laboratory teaching as well as the supervised clinical training and shifted to alternative methods of teaching delivery and assessment such as online lectures, webinars, problem-solving sessions, written reports and computer-based exams. With modern technology at our fingertips, it is practical for the students to access the contents of each lecture from home and avoid unnecessary attendance at the lectures than can increase the risk of spread of infection. E-learning in some way encourages self-learning independency amongst the students and improves their ability to use online resources [76].

It should be noted that the students can get very stressed during COVID crisis and become negatively impacted by the fear of getting infected by the virus and hence the need for counselling services and psychological support may increase following COVID-19 pandemic [77]. A recent publication in the British Dental Journal highlighted the thoughts of the Dean of the dental school in Queen Mary University of London regarding COVID-19 outbreak and his management approach at the time of crisis. It was mentioned that initiative was taken based on the moral judgement to stop all patient care for undergraduate and postgraduate clinics to save lives of students, staff and patients, which in turn encouraged further discussion on this topic and changed the situation in the critical time of pandemic [78].

### The Role of Simulation in Dental Education 

Due to a high risk of COVID-19 transmission in dental clinics and hospitals, alternative modes of teaching have been explored. Simulation exercises are one of the safest forms of clinical skills practice without the need for physical presence in the clinical environment and direct contact with patients. The main target of dental education is to train independent dentists who are able to treat their patients effectively and safely. Therefore, students must have excellent manual dexterity with fine motor skills. These qualities can be developed and acquired in simulation settings during undergraduate training. Teaching these skills to the required standard is a real challenge considering the fact that allocated time and resources are not unlimited [79,80]. Simulation has also been used to facilitate the transition into the dental clinic and enhance a student’s preclinical experience through inclusion of a wide range of simulated patient scenarios and exercises [81]. The recent advancement in virtual reality (VR) simulation technology brings a range of opportunities for education in dental schools. It provides both students and the tutor with an integrated on-screen continuous feedback on performance of the trainee [82]. The combination of recent haptic technology has equipped the VR simulators with the ability of tactile feedback that makes it possible for the trainee to feel and touch the virtual tooth tissue. There is evidence suggesting that the use of VR technology improved the rate of skill acquisition in operative dentistry modules taught in undergraduate dental programs [83]. Research has shown that a VR simulation facility alongside an experienced instructor providing real-time feedback is the most productive method of teaching in a simulation environment. It improves students’ hand-eye coordination, fine motor skills and reflection skills and becomes especially effective in the very early stages of skill acquisition leading to a conservative preparation approach and better skill retention [84,85,86]. Thus, it can be proposed that VR simulation technology is a useful supplementary tool to conventional dental training approaches, and its effective and safe use possibly with some modifications to allow distance learning can be considered during COVID-19 pandemic.

## 8. Impact of COVID-19 on Dental Research 

The immediate impact of COVID-19 pandemic on dental research was noticed upon cancellation of the International Association of Dental Research (IADR) conference in Washington DC, USA, in March 2020, which is one of the largest international dental research events. Many clinical dental research studies were impacted by the COVID-19 outbreak because of the need for social distancing and health authorities’ guidance to stop non-urgent dental procedures and outreach activities. In addition, most laboratory-based dental research projects and post-graduate student research projects were suspended due to mandatory government and institutional policies limiting non-essential research activities. Therefore, some dental researchers shifted their focus to off-campus and online means of research such as conducting online surveys and carrying out literature reviews. 

Priority was given particularly to COVID-related research, and the funding bodies announced various calls for research and development in the field of COVID-19.

Potential areas for future dental research relevant to the COVID-19 outbreak may include but are not limited to the following categories:

- Dental Public Health issues in relation to COVID-19.
- Impacts of the novel coronavirus pandemic on dental professionals and the dental sector.
- Cross infection control and PPE in dentistry.
- Role of dental professionals and patients in screening, prevention, diagnosis and management of the novel coronavirus disease.
- Innovations in remote consultation and alternative IT-based methods in dental education and training
- Oral biology research in terms of the interactions of human oral tissues with COVID-19 and the effects of COVID-19 infection or its treatment on oral hard and soft tissues. Application of tissue engineered models of human oral and respiratory mucosa in this field can be a clinically relevant and rapid tool in investigating the disease mechanisms and potential therapeutic approaches [87,88].

## 9. The Role of Tele-Medicine in Controlling the Pandemic 

Many experts in healthcare communications and telemedicine technology believe that the newly emerged service of Telehealth is a great tool to close the distance between the public, doctors and healthcare providers. It enables everyone to stay in their houses while holding communication with their physician through a virtual channel, contributing to slowing down and reducing the virus spread to mass population and the medical team [89]. There are many hospitals across the US and Europe benefiting from telehealth technology to treat their quarantined patients and those infected by COVID-19. By adopting telehealth facilities, patients who suffer from other medical conditions and chronic diseases can receive the appropriate care via on-line platforms without the need to enter the medical facilities, which minimizes the risk of contracting the virus. It can also be used for the initial triage of patients prior to their arrival at hospitals and dental clinics [90].

### 9.1. Minimising the Risk to Health Care Workers 

In order to increase the safety of medical team in the frontline, risk assessment should be carried out for all patients and proportionate measures must be taken for each patient risk category. Most patients with chronic conditions who need repetition of their prescription will significantly benefit from telehealth to reduce the risk of exposure to COVID-19. Hospital doctors and specialists who require to self-isolate would also benefit from telehealth facilities to see the patients remotely and help the healthcare system when the resources are stretched [90].

The main limitation of this method of healthcare provision is that most hospital and private practices are not equipped enough to deliver telehealth services. There is also need for staff training, which requires time and adaptation to the novel system. The lack of suitable technical and network infrastructures as well as specialized hardware is also an issue that needs to be considered and addressed for the future. A survey in the USA showed that 84 percent of the population tend to choose a healthcare provider with telemedicine on offer over the ones that do not offer it, and it appears that patients are developing a tendency towards modern tech-based healthcare [90].

### 9.2. Volunteer and Support 

It is important to note that the amount of support from ordinary people and active and retired healthcare professionals who volunteered to be deployed across the healthcare networks in the UK and overseas was incredibly overwhelming. These invaluable highly-skilled volunteers should be professionally organized and registered for the future crisis should it happen. 

## 10. Potential Long-Term Impact of COVID-19 on Dentistry 

Authors’ observations and evaluation of the current situation suggest that the costs of providing dental treatment may increase in the future because of several reasons including the need for additional resources such as PPE, dental practice modifications and increased waiting times due to the need for segregation of patients in the waiting areas resulting in a reduced number of patients that can be seen daily. Additionally, due to an increased occupational health risk to the practitioners arising from carrying out AGPs, the cost of specialist services may possibly increase. It is also assumed that there may be a surge in the demands for e-consultations in the near future. 

There could be a general fear of visiting dentists among the public after the COVID-19 outbreak settles down. Consequently, demand for the elective dental treatments could decrease, and patients may choose emergency extractions over preservative treatments such as root canal treatment. On the other hand, to avoid and prevent dental problems, some patients will pay more attention to their oral and dental health by improving their oral hygiene practice and following preventative recommendations. It is also expected that social distancing, self-isolation and quarantine during COVID-19 pandemic may lead to an increase in the risk of mental health disorders, cardiovascular disease due to reduced mobility and increased risk of other medical conditions such as diabetes, which may subsequently influence patients’ dental health and have significant implications for dentistry in terms of the role of patient’s medical history, which will require further attention in the future.

Due to overall economic impact of COVID-19, extended lockdown measures and closure of dental surgeries, it is predicted that there could be further uncertainty for the profession, reduced incomes and more job loss in the future. Some small to medium-size dental related businesses may not survive a long-term lockdown. A recent poll [91] of 2860 UK dental practices (24.3% of around 11,800 practices) revealed that 71.5% of the practices could sustain their business finances for 3 months or less. A total of 20% of practices had an estimation that with current situation, their businesses could survive only until the end of April 2020. A total of 75% of practices with greater proportion of private work seemed to be more vulnerable to the current economic climate and believed that they would experience forthcoming hardship in the next three months. The chair of British Dental Association stated that the decision to suspend all non-emergency dental treatment was correct; however, dental services in the UK would face devastation without significant financial support [91]. In the UK, contracted NHS practices received significant government funding during the COVID-19 outbreak, although there are some concerns regarding sustainability of the NHS contracts in their existing forms following the pandemic. The private sector, which accounts for 50% of the dental economy in the UK, has received very little revenue except for some small grants and loans, making the effect of COVID-19 devastating for the private sector. Some credit rating agencies have changed investor rating of dental companies to negative in April 2020, expecting a huge reduction of 50% in volume of dental patients for the financial year ending 31 March 2021. If these speculations materialize in the future, most of the private dental practices will experience a reduction of 66% in their profit or will not be able to make a profit at all [92]. In addition, the uncertainty about the long-term behavior of the virus makes it very difficult to introduce a practical and long-lasting plan to tackle the current crisis. On 14 May 2020, the executive director of the WHO’s health emergencies program stated that there is a chance that the novel coronavirus can never be eradicated as the HIV has not been yet. 

A long period of uncertainty could encourage some dentists to decide to take their pensions and become retired. That could have a significant effect on the market and affect the value of dental practices. Many patients may also expect a payment refund, and this could have a negative financial impact on many practices. There is also no guarantee that patients will return to complete their initiated treatments and indeed may stop being regular attendees. Another possibility is that due to difficulties associated with dentist visits, some patients with infected gums and teeth, which have remained untreated, could develop serious long-term dental infection-related complications. It is also expected that due to suspensions of conservative restorative treatments, more teeth could be extracted, and therefore, there will be an increased demand for replacement of missing teeth with simple cost-effective removable prosthetic treatment with high patient satisfaction [93] in the short-term and the possible need for fixed tooth-retained and implant-retained prosthesis in the long-term. 

There is a speculation that COVID-19 outbreak may encourage the use of digital intraoral scanners instead of conventional impressions and may increase the demand for computer aided design and manufacturing (CAD/CAM) and 3D-printing technology. Thus, there could be an increasing demand for more productive and cost-effective digital dentistry equipment, which in turn may boost the industrial aspect of dentistry and flourishing engineering potentials by further investment from main stakeholders and investors. 

With regards to the long-term effect of the COVID-19 outbreak on the dental education, it has negatively affected face-to-face teaching and clinical supervision, and therefore, there could be a reduction in the number of applicants for undergraduate and postgraduate dental programs in the future. However, this current pause on conventional educational activities creates an opportunity for alternative methods of teaching to be tested and improved, and it will encourage more creativity amongst education providers resulting in introduction of novel means for clinical and face-to-face teaching. It is also important to mention that the total number of university applications from international students has also experienced a significant reduction. This can create a financial ambiguity for the universities and institutions, which used to be an attractive hub for international students for years.

It is predicted that there would be reduced funding for dental research due to economic impact of COVID-19 leading to a decline in the clinical and laboratory-based dental research activities. However, there could potentially be increased funding for COVID-19 related dental research due to the need for new knowledge and discovering ways to face this challenge. 

## 11. Re-Opening

Dental practitioners, educators and researchers must follow regulations and guidelines announced by their local authorities and institutions as how to operate post COVID-19 outbreak. It is expected that a robust operational protocol in workplace would be an essential element of preparations for re-opening of dental services. An example of such protocol has been released by the European Federation of Periodontology (EFP) [94], which provides dental practitioners with a patient management tool. The infographic documents suggest that all the patient should undergo an initial phone triage in order to assess the patient’s risk profile and needs so that the dental team can organize the clinical agenda and waiting lists accordingly. The EFP also suggested a strict protocol on patients’ arrival and additional PPE for both patients and the dental team. It recommended that disinfection of the working field via mouth rinse should be carried out in addition to utilizing high-volume suction under rubber dam isolation of the teeth during all AGPs and careful doffing of all PPE after each visit [94]. British Association of Private Dentistry (BAPD) published a position paper in May 2020 regarding return to dental practice after COVID-19 [92]. They advised practitioners to carefully look at the scientific evidence and avoid purchasing expensive equipment without a sound evidence-base. They also recommended preparation of practices for social distancing and minimal contact patient flow as well as ensuring sufficient PPE is available. Furthermore, a rapid review of internationally produced guidance for re-opening dental services was published by Cochrane oral health [95] to support decision making on planning the re-opening and restructuring of dental services. This rapid review collated and summarized recommendations from the various sources identified within five themes relevant to the re-opening of dental services including practice preparation, personal protective equipment, management of the clinical area, dental procedures and cleaning and disinfection [96]. On 12 May 2020, the national UK dental organizations joined forces agreed on a return to work guidance within the COVID-19 Future Planning Task Group after COVID-19 outbreak eases [97]. 

As for dental education, the authors believe that it would be beneficial to switch the seminars and lectures to online platforms to avoid unnecessary gathering of students and maintain the social distancing principles. There also should be a development plan in place for further integration of more interactive digital learning platforms for delivering the modules in dental education. The operative skills acquisition of students is the milestone in training the future generation of the dentists, and therefore, evidence-based alternative methods of teaching such as VR would be useful, which in turn provides the learners with a safe and feasible environment to advance their basic operative skills and manual dexterity. Non-clinical teaching can be prioritized and delivered as much as possible during the COVID-19 outbreak until the clinical teaching facilities can re-open. Managing the undergraduate and postgraduate student teaching clinics requires a joint effort by the universities and the teaching hospitals to develop and organize an efficient system for re-opening and implementation of the relevant guidelines and regulations. 

In terms of dental research, the laboratory-based routine dental research usually does not require direct patient contact and can be resumed more swiftly following COVID-19 lockdown compared to the clinical dental research. The decision on the type of research that can take priority will depend on the strategic organizational research priorities. Research activities will require thorough risk-assessment and must follow the relevant health and safety regulations including the PPE requirements as well as the local social distancing policies. 

## 12. Conclusions and Future Trends 

In conclusion, COVID-19 has had many immediate complications for dentistry of which some may have further long-term impacts on clinical practice, dental education and dental research. It is important to consider the following points in the long-term:

- Preparedness and contingency planning for modifying clinical practice in dentistry.
- Optimization of cross-infection control protocols.
- Further focus on prevention and oral health promotion for the public.
- Patient empowerment and education.
- Incorporation of modern IT-based and online forms of teaching and assessment into dental education, which can also help the environment and reduce pollution.
- Increased role of e-consultancy and tele-medicine.
- Further investment in relevant dental research fields.
